# The Moderating Role of Family Functionality in Prosocial Behaviour and School Climate in Adolescence

**DOI:** 10.3390/ijerph20010590

**Published:** 2022-12-29

**Authors:** Alba González Moreno, María del Mar Molero Jurado

**Affiliations:** Department of Psychology, University of Almeria, 04120 Almeria, Spain

**Keywords:** family environment, social behaviour, school, secondary education, adolescence

## Abstract

Adolescence entails a series of changes in young people who need to adopt socially positive behaviours and have a beneficial family and school environment. The main objective of this research is to analyse the relationships established between the variables of prosocial behaviour and school climate, as well as to check whether family functionality plays a moderating role in this relationship. The participants were a total of 743 adolescent students between 14 and 19 years of age from different schools in the province of Almería (Spain). The instruments used to evaluate the young people were the APGAR family functioning scale, the Prosocial Behavior Questionnaire (CCP) and the School Social Climate Questionnaire (CECSCE). The data analysed showed a positive correlation between all the variables analysed: family functioning, prosocial behaviour and school climate. Gender differences were found, with adolescent girls showing higher levels of empathy and respect, while boys scored higher in social relations and school climate. The results indicate that family functioning plays a moderating role in some dimensions of prosocial behaviours and school climate. The importance of attending to these types of variables in adolescent students to foster optimal youth development and promote their personal well-being is discussed.

## 1. Introduction

Adolescence is explicitly characterised as a developmental process in which young people begin their independence and identity in different aspects of life such as physical, sexual, emotional and psychosocial [1]. This transitional stage between childhood and adulthood brings with it a number of risk factors that can lead to problems that have serious consequences for the rest of life [2], such as drug addiction [3] or the development of drug use disorders [4,5]. This period stands out for the vulnerability of young people and their desire to experience new sensations together with social influences and peer pressure [6]; the most commonly used coping strategies are avoidance and social support [7]. This search for new sensations may lead adolescents to engage in activities that involve risky behaviour, such as drug use [8], perpetuation of violence [9] or unsafe sexual behaviours, which have negative consequences such as sexually transmitted infections or unwanted pregnancies [10]. These risky behaviours can cause mental or physical harm and, if not identified early, young people’s health and their family and social bonds can be severely damaged [11,12]. In recent years, research on problems affecting young people has shifted from focusing on aspects of individual development to the impact of social contexts [13]. Various theoretical models focused on this social influence paradigm provide an explanation for these types of behaviours. Bandura and Walters’ theoretical foundation on social learning states that all human behaviour is learned by imitation through observation [14]. On the other hand, Bronfenbrenner’s Ecological Theory argues that individual behaviours are influenced by young people’s own social environment, such as family and school [15].

The family is the place where the adolescent develops from birth; it is the main nucleus in covering the basic needs of adolescents and imparting a series of norms, beliefs and traditions that are necessary to develop in society [16]. Therefore, the functionality of the family has an impact on the adaptation, life satisfaction, coping and, consequently, the well-being of the individual [17,18]. Family functioning is one of the most relevant factors that serve to assess the quality of the family environment, communication, coping and conflict resolution, emotional response, established roles and behavioural control among family members [19]. The benefits of positive family functioning in adolescence are reflected in current studies, which indicate that students with highly functioning families are reported to have greater social support than young people with dysfunctional families [20]. Furthermore, satisfaction with family life is estimated to be a mediator of the relationship between social connectedness and problematic social network use [21].

Regarding family functioning and its relationship with adolescent behaviour, recent studies indicate that a dysfunctional family environment is associated with negative behavioural and health outcomes for adolescents [22]. Similarly, it can be indicated that family functioning acts as a predictor of the onset of substance use, such as alcohol or tobacco, as a high score on family functioning is significantly related to the lower consumption of these substances [23]. The current scientific literature focuses on identifying the relationships between family functioning and risk behaviours in adolescents [24,25]. With regard to prosocial behaviour, it should be noted that it refers to a type of attitude in which people, voluntarily and without the purpose of obtaining a benefit in return, offer help, share and comfort others [26]. One of the dimensions of prosocial behaviours, such as leadership, has been shown to correlate positively with behaviour-oriented strategies, as well as interpersonal relationships with peers [27]. Furthermore, this relationship between prosocial behaviours and personality traits has also been found, as individual differences in prosocial behaviour are the result of traits that are expressed in response to certain interdependent situations [28]. Referring to the previous literature, the first part of the hypothesis of our study has been formulated, which states that there is a significant relationship between family functioning and prosocial behaviours. However, one aspect to consider is that research focused on prosocial behaviours is scarce, although there are programmes that show that students who increase their antisocial behaviours show worsening prosocial behaviours [29]. There is a need to promote prosocial behaviours in young people, as these skills are related to personal well-being and self-esteem [30]. The development of prosocial behaviour may differ according to gender [31]. According to gender socialisation theory, girls tend to be educated to show care and affection, while boys tend to inhibit this type of prosocial behaviour [32]. Studies focusing on such differences in adolescents reveal that boys report lower levels of prosocial behaviour compared to girls [33]. All these studies have led to the second hypothesis of the study, which states that there are gender differences in students’ prosocial behaviour.

Regarding family functioning and school climate in adolescents, it is worth noting that several studies point out that young people from dysfunctional families have greater problems with school attendance and school refusal [34]. School climate is understood as the character and quality of school life that reflects the established norms, values, goals, teaching practices and interpersonal relationships that are established in the educational context [35]. Family functioning is thought to act as a moderating factor in adolescents’ perceptions of classroom climate, as a significant effect has been found for participants with high versus low family functioning [36]. The importance of fostering school climate is shown in studies indicating that high levels of responsibility correlate significantly with high levels of school social climate and low levels of violence [37]. Based on the studies examined, the second part of the first hypothesis of our study is composed, where the existing relationships between family functionality and school climate are tested. Regarding the differences found according to gender, it is worth noting that adolescent girls have higher academic expectations, as well as higher scores in academic stress and emotional problems than boys [38,39]. Thus, based on this research, the second hypothesis of this study focuses on the differences according to sex in the perception of school climate. All these positive effects of family functioning and its relationship with prosocial behaviour and school climate have given rise to the third hypothesis of the study, where it is estimated that family functioning acts as a moderator of school climate and prosocial behaviour in adolescents.

### Objetive and Hypothesis of the Study

The aim of this research is to analyse the relationships established between the variables of prosocial behaviour and school climate, as well as to check whether family functionality plays a moderating role in this relationship. The initial hypotheses previously argued that have been considered for this study are the following:

**Hypothesis 1 (H1).**  *Family functionality relates positively to prosocial behaviours and the school climate, as well as the latter two to each other.*

**Hypothesis 2 (H2).**  *There are sex differences among adolescents in the variables examined.*

**Hypothesis 3 (H3).**  *Family functionality acts as a moderator of prosocial behaviours and school climate in adolescents.*

## 2. Materials and Methods

### 2.1. Study Design and Participants

This quantitative study was based on a cross-sectional descriptive design and, therefore, followed the STROBE guidelines for cross-sectional studies [40]. The sample consisted of 743 students of Compulsory Secondary Education (ESO) from six schools in different regions of Almería (Spain). Participants were between 14 and 19 years old (*M* = 14.99; *DT* = 0.86), with 50.7% women (*n* = 377) and 49.3% men (*n* = 366). Overall, 57 per cent of these students were in third grade and 49.1 per cent in fourth grade. Regarding nationality, most of the participants were of Spanish descent (92.9%), although students of other ancestries such as Moroccan, Colombian, Romanian or Venezuelan also participated.

### 2.2. Instruments

A booklet containing the instruments already validated by other researchers was prepared by the authors together with an ad hoc questionnaire that revealed sociodemographic aspects of the participants such as sex, age, academic year or nationality. 

Family Functionality (APGAR) [41]. The Spanish adaptation of the original Family Functionality Scale has been used [42]. This self-report scale evaluates perceptions of family functioning by exploring people’s satisfaction with their kinship. The instrument consists of five items, answered on a three-point Likert scale, ranging from 2 (almost always) to 0 (almost never). Each item measures one of the five dimensions: (1) Adaptation (family resources available to deal with situations of stress or periods of crisis; for example, “When something worries me, I can ask my family for help”); (2) Participation (involvement of members in sharing family issues and decisions; for example, “I like the way my family talks and shares problems with me”); (3) Personal resource gradient (support and acceptance of changes in individual family members; for example, “I like how my family allows me to do new things I want to do”); (4) Affection (emotional expressions and affection between family members; for example, “I like what my family does when I’m sad, happy, upset, etc.”); (5) Resources (commitment to spending time and meeting the emotional and physical needs of other family members; for example, “I like how my family and I share time together”). The interpretation of this scale is made based on the total score obtained, taking into account the following classification: normal functionality of 7–10 points, moderate dysfunction of 4–6 points and severe dysfunction of 0 to 3 points. The internal consistency achieved with this instrument was good (*α* = 0.84).

Prosocial Behaviour (CCP) [43]. Prosocial behaviour was evaluated through the Prosocial Behavior Questionnaire aimed at children and adolescents between 10 and 17 years old, evaluating the different behaviours of help used by participants, such as sharing, understanding, encouraging and collaborating. It consists of a total of 55 items, divided into four components, and has four response options ranging in a Likert scale from “never” to “always”. The first factor called Empathy consists of 20 items and refers to the person’s ability to put himself in the place of the other and relieve his discomfort (e.g., “When someone has problems I worry”). The second factor is named Respect; it groups 16 items and refers to the ability to treat others assertively and politely (e.g., “When I offend or upset, I apologize”). The third factor is called Social Relations, which consists of 11 items and deals with the ability to carry out positive social relations (e.g., “I like to talk with my friends and colleagues”). Finally, the fourth factor is Leadership, which consists of eight items and refers to the ability to lead and organise group activities (e.g., “I like to lead group work”). Acceptable and excellent results in internal consistency were obtained for each of these dimensions: Empathy (*α* = 0.90), Respect (*α* = 0.78), Social relations (*α* = 0.69) and Leadership (*α* = 0.74).

School Climate (CECSCE) [44]. The Social Climate Questionnaire of the School is a validated instrument in the Spanish adolescent population that was elaborated from the items of the California School Climate and Safety Survey [45,46]. This questionnaire consists of a total of 14 items that are answered using a Likert scale from 1 (never) to 5 (always) and is composed of two factors. The first factor refers to the climate of the school (e.g., “Students really want to learn”) and their score is obtained by adding the answers to their items. The second factor indicates the climate regarding the teaching staff and addresses the perceptions that students have about the performances of the teaching staff (e.g., “Teachers at this school are pleasant with their peers”), and their score, as in the previous factor, is obtained by adding the evaluation of the responses collected. The internal consistency obtained was acceptable both in the school climate factor (*α* = 0.79) and in the teacher climate factor (*α =* 0.77). 

### 2.3. Procedure

Once the booklet was prepared with the instruments of the variables that were to be examined, several educational centres of different municipalities in the province of Almería were contacted. A total of six secondary schools agreed to participate in this study, so it was agreed with the management of the school one day to attend and the students completed the booklet in person. Before starting the data collection, all students and their legal guardians were informed of the purpose of the study and gave their consent to participate in it. The data collection was carried out during the months of February to June 2022. The research was approved by the Committee of Bioethics in Human Research of the University of Almería with reference UALBIO2021/025.

### 2.4. Analysis of Data

After the collection of all data, they were recorded for further analysis in the statistical analysis program SPSS version 28 [47]. The reliability of the instruments used was examined using the Cronbach alpha coefficient, which stipulates the following general rule for interpreting the results: <0.5 unacceptable, >0.5 poor, >0.6 questionable, >0.7 acceptable, >0.8 good and >0.9 excellent [48].

A descriptive analysis was carried out in order to provide relevant information about the students who participated in this study. In addition, a Pearson’s bivariate correlation analysis was carried out to determine whether there is an association between the variables studied. The absolute values obtained were interpreted according to the following categories: no correlation between 0 and 0.10, weak correlation between 0.10 and 0.29, moderate correlation between 0.30 and 0.50 and, finally, strong correlation between 0.50 and 1.00 [49]. 

The Student’s *t*-test for independent samples was then calculated. This test was carried out to examine the differences between adolescent girls and boys in the variables analysed and their corresponding dimensions. Cohen’s d was calculated to estimate effect sizes: <0.50 small, 0.50–0.80 medium and ≥0.80 large [50].

Subsequently, in order to examine the behaviour of family functioning as a moderating variable of prosocial behaviours (empathy, respect, social relations and leadership) as predictors of school climate dimensions (school and teacher), simple moderation analyses were carried out. For this, we used the medmod module integrated in Jamovi v.2.3.2 [51], which allows the computation of models on simple moderation effects, providing information on standardised coefficients, z-scores and significance levels. The bootstrapping technique was applied with estimated coefficients from 5000 bootstrap samples, with a 95% confidence interval.

## 3. Results

### 3.1. Descriptive Analyses and Correlations

The results obtained show how the variables examined correlate with each other (Table 1). 

Family Functionality is positively related both with the dimensions of Prosocial Behaviour: Empathy (*r* = 0.15; *p* < 0.001), Respect (*r* = 0.25; *p* < 0.001), Social Relationships (*r* = 0.31; *p* < 0.001) and Leadership (*r* = 0.26; *p* < 0.001); and with School Climate: School Climate (*r* = 0.41; *p* < 0.001) and Teacher Climate (*r* = 0.31; *p* < 0.001). 

These data are similar to those obtained with the Prosocial Behaviour and School Climate variables, due to the fact that both constructs show a positive correlation between them. As for the School Climate dimension, the scores for Prosocial Behaviour are as follows: Empathy (*r* = 0.29; *p* < 0.001), Respect (*r* = 0.34; *p* < 0.001), Social Relations (*r* = 0.31; *p* < 0.001) and Leadership (*r* = 0.22; *p* < 0.001). 

As mentioned above, these positive correlations are also found with the other dimension of School Climate called Teacher Climate: Empathy (*r* = 0.33; *p* < 0.001), Respect (*r* = 0.41; *p* < 0.001), Social Relationships (*r* = 0.24; *p* < 0.001) and, finally, Leadership (*r* = 0.18; *p* < 0.001).

### 3.2. Gender Differences in the Variables Examined

The data in Table 2 show the existence of differences according to gender in different dimensions of Prosocial Behaviours and perceptions of School Climate. On the other hand, no significant differences were found between the two groups in the Family Functionality variable.

Referring to the Prosocial Behaviours variable, it is worth noting that adolescent girls have a higher score in Empathy (*t* = −6.24; *p* < 0.001; *d* = 0.46) and Respect (*t* = −2.45; *p* < 0.05; *d* = 0.18), while boys stand out in Social Relationships (*t* = 2.30; *p* < 0.05; *d* = 0.17). 

As for the perception of school climate, the results obtained indicate that boys have a higher level in the dimension of School Climate (*t* = 0.42; *p* < 0.05; *d* = 0.03) compared to girls. No differences were found in the perception of Teacher Climate between the sexes.

### 3.3. The Moderating Effect of Family Functioning on the Predictive Value of Prosocial Behaviours in the School Climate with Respect to the School and Teachers

**Figure 1:**
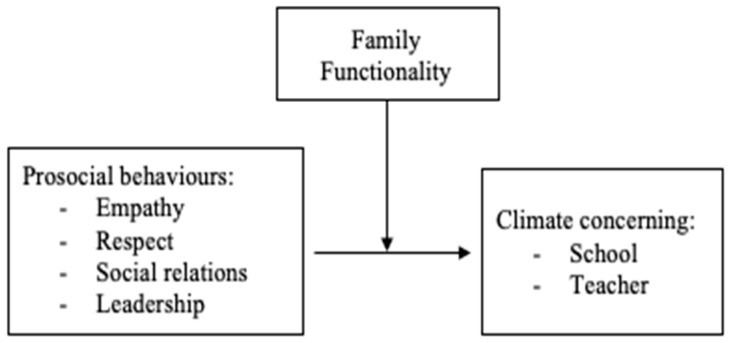
Hypothetical model of the moderation of Family Functionality in the relationship between Prosocial Behaviour and School/Teacher Climate.

Based on the simple moderation models, the coefficients of the effects of each of the independent variables (cognitive empathy and affective empathy), of the moderating variable (family functioning) and of the interaction term on the dependent variables (perception of school climate in relation to the school and in relation to the teacher) are estimated in each case. Figure 1 shows the proposed theoretical model graphically, with the variables involved in the simple moderation analyses.

In the first analysis, Prosocial Behaviours were included as a predictor variable in each case, and School Climate as a criterion variable, considering the moderating role of family functionality. In all cases, prosocial behaviours establish a positive and significant relationship with school climate: Empathy (*β* = 0.13; *SE* = 0.018; *z* = 7.28; *p* < 0.001), Respect (*β* = 0.20; *SE* = 0.028; *z* = 7.02; *p* < 0.001), Social Relations (*β* = 0.25; *SE* = 0.041; *z* = 6.05; *p* < 0. 001) and Leadership (*β* = 0.15; *SE* = 0.043; *z* = 3.50; *p* < 0.001).

In addition, the results report a moderating effect of family functioning for centre climate: Empathy * Family Functioning (*β* = 0.02; *SE* = 0.006; *z* = 3.22; *p* < 0.001), Respect * Family Functioning (*β* = 0.02; *SE* = 0.009; *z* = 2.48; *p* < 0.05) and Social Relations * Family Functioning (*β* = 0.03; *SE* = 0.013; *z* = 2.60; *p* < 0.01). For the climate referring to the centre, the moderating effect of Family Functioning was not verified taking Leadership as a predictor variable (*β* = 0.16; *SE* = 0.013; *z* = 1.15; *p* = 0.250).

For the second analysis, Prosocial Behaviours were included as predictor variables and Teacher Climate as a criterion variable, again considering the moderating role of Family Functioning. In all cases, Prosocial Behaviours were positively and significantly related to Teacher Climate: Empathy (*β* = 0.13; *SE* = 0.016; *z* = 8.41; *p* < 0. 001), Respect (*β* = 0.23; *SE* = 0.025; *z* = 9.46; *p* < 0.001), Social Relationships (*β* = 0.15; *SE* = 0.038; *z* = 4.11; *p* < 0.001) and Leadership (*β* = 0.10; *SE* = 0.039; *z* = 2.61; *p* < 0.01).

In addition, the data revealed a moderating effect of Family Functioning for Teacher Climate, taking Empathy as a predictor variable (Empathy * Family Functioning; *β* = 0.01; *SE* = 0.005; *z* = 2.37; *p* < 0.05). In this case, for the climate referring to the centre, the moderating effect of Family Functionality was not confirmed taking as predictor variables: Respect (Respect * Family Functioning; *β* = 0.005; *SE* = 0.008; *z* = 0.62; *p* = 0.533), Social Relations (Social Relations * Family Functioning; *β* = 0.01; *SE* = 0.011; *z* = 1.66; *p* = 0.098) and Leadership (Leadership * Family Functioning; *β* = 0.006; *SE* = 0.012; *z* = 0.55; *p* = 0.582).

## 4. Discussion

This research was able to respond to the objectives initially set out, so that the different relationships existing between the variables analysed (family functionality, prosocial behaviour and school climate), the differences according to sex and whether family functionality acts as a moderating factor in the prosocial behaviour and school climate of adolescent students have been verified. 

The first hypothesis proposed was corroborated by the results obtained. The data collected indicate that there is a positive correlation between family functioning and prosocial behaviour. This idea is linked to the idea that the family is the main nucleus of young people’s development, where a series of social norms, behavioural control and, consequently, adolescents’ own well-being are established [14,15,16,17]. Thus, as other studies have shown, positive family functioning is beneficial in adolescence as it mediates the problematic use of social networks and increases perceived social support [18,19]. There is a need to promote prosocial behaviours in order to reduce disruptive and health-risk behaviours in young people and increase their self-esteem and personal well-being [7,8,9,10,27,28]. Such a positive relationship has also been found between family functioning and school climate, which is linked to subsequent studies indicating that young people from dysfunctional families have problems at school [32]. 

The second hypothesis was confirmed in two of the three variables analysed, specifically, differences were found in relation to gender in prosocial behaviours and the perception of school climate among adolescents. With regard to prosocial behaviours, it was found that girls report higher scores in different dimensions such as Respect and Empathy compared to boys. Previous research affirms that adolescent boys perform fewer prosocial behaviours than girls [31], thus corroborating the extracted data. These results may be related to gender socialisation theory, which indicates that there may be differences in the development of prosocial behaviour [30]. Both sexes are educated differently when it comes to expressing their feelings or behaving [29]. On the other hand, in relation to the perception of the school climate, it was found that boys rate the school climate more positively than girls. This statement may be linked to the fact that, as shown in previous studies, adolescent girls have higher academic stress and school expectations than boys [36,37]; it is possible that these factors modify girls’ perception of school climate in a less positive way than boys. Finally, no significant differences were found between adolescents regarding family functioning.

The third hypothesis focused on family functionality acting as a moderator of prosocial behaviours, and the perception of school climate in adolescents was also confirmed. The analyses carried out show that the relationship between prosocial behaviours of empathy, respect and social relations and the school climate is moderated by family functioning. This statement is associated with other studies indicating that family functioning affects adolescents’ behaviour both outside and inside the school environment [20,21,22,23]. However, this moderation in leadership is not found, which may be related to the fact that this dimension of prosocial behaviours depends more on factors such as interactions among the class group than on family functionality [25]. On the other hand, in the relationship between prosocial behaviours and teacher climate, only the association with Empathy is moderated by family functioning. These results may be linked to the fact that prosocial behaviours interact with family functioning in relation to school climate rather than teacher climate [34]. 

## 5. Conclusions

In conclusion, it is worth highlighting how this research has allowed us to learn about different aspects of family functionality, prosocial behaviour and school climate. The practical implications of this research are to delve deeper into variables that are immersed in adolescence, as this is a stage of many changes that can alter the proper development of young people. Some of the limitations to be highlighted are that previous literature focuses more on antisocial or risky behaviours that occur in adolescence than on positive behaviours such as prosocial behaviours. Therefore, it would be interesting to consider, as a future line of research, which variables enhance the correct development of adolescents in society in order to promote these constructs and reduce risk factors and increase their personal wellbeing. The need to attend to elements such as family functionality, school climate or prosocial behaviours with the aim of promoting the personal well-being of adolescents is highlighted.

## Figures and Tables

**Table 1:** Descriptive data and correlation matrix between Family Functionality, Prosocial Behaviour and School Climate (*N* = 743).

	APGAR	Empathy (CCP)	Respect (CCP)	Social Relationships (CCP)	Leadership (CCP)	School Climate (CECSCE)	Teacher Climate (CECSCE)
APGAR	-						
Empathy (CCP)	0.15 ***	-					
Respect (CCP)	0.25 ***	0.53 ***	-				
Social Relationships (CCP)	0.31 ***	0.39 ***	0.28 ***	-			
Leadership (CCP)	0.26 ***	0.35 ***	0.16 ***	0.53 ***	-		
School Climate (CECSCE)	0.41 ***	0.29 ***	0.34 ***	0.31 ***	0.22 ***	-	
Teacher Climate (CECSCE)	0.35 ***	0.33 ***	0.41 ***	0.24 ***	0.18 ***	0.65 ***	-
Media	6.64	55.98	48.91	32.78	20.57	24.37	19.49
SD	2.90	9.62	6.62	4.66	4.52	5.38	4.55
Min.	0	21	27	19	8	9	6
Max.	10	75	77	44	32	40	30

*** *p* < 0.001; APGAR = Family Functionality Scale; CCP = Prosocial Behaviour Questionnaire; CECSCE = School Social Climate Questionnaire.

**Table 2:** *T*-test by sex (girls *n* = 377; boys *n* = 366).

		Sex				*t*	*p*
		Boys		Girls			
		Media	SD	Media	SD		
Family Functionality Scale (APGAR)	Total APGAR	6.69	2.88	6.60	2.92	0.43	0.665
Prosocial Behaviour Questionnaire (CCP)	Empathy	53.80	9.67	58.10	9.08	−6.24 ***	<0.001
	Respect	48.30	7.07	49.49	6.11	−2.45 *	0.014
	Social Relationships	33.18	5.03	32.39	4.24	2.30 *	0.021
	Leadership	20.70	4.62	20.44	4.43	0.77	0.439
School Social Climate Questionnaire. (CECSCE)	School climate	24.46	5.38	24.29	5.39	0.42 *	0.021
	Teacher climate	19.17	4.70	19.79	4.39	−1.85	0.439

* p < 0.05; *** *p* < 0.001.

## Data Availability

Not applicable.
